# Hospital and Institutional News

**Published:** 1913-04-26

**Authors:** 


### HOSPITAL AND INSTITUTIONAL NEWS.

### THE YORK COUNTY HOSPITAL COMMISSION

We publish this week a full verbatim report of the proceedings before the Special Commission, of which Sir Cooper Perry, M.D., is President, which sat at the York County Hospital, on Friday and Saturday, 18th and 19th instant, to take evidence and go into the charges. The proceedings lasted from ten in the morning to six in the evening each day. We believe that those who begin to read this report will undoubtedly read it to the end. It covers much ground, and many of the points raised must prove of value to all interested in the hospitals. Sir Cooper Perry, in due course, will report to the York Hospital authorities. The publication of the evidence will bring many questions of hospital administration to the notice of the whole hospital world. Our columns are open to all correspondents, for their contributions, if based upon knowledge and experience, must have an educational value.

### KING EDWARD'S HOSPITAL FUND FOR LONDON.

The annual meeting of the Governors and General Council of this fund was held in the picture gallery of St. James's Palace on the 23rd instant, the Speaker of the House of Commons in the chair. The account of the proceedings reached us too late to enable us to publish it in full in the present issue. The account of receipts and expenditure for 1912 shows total receipts amounting to £179,379. The amount distributed to hospitals was £151,000, and to convalescent homes £6,500, making a total of £157,500, which entailed a payment from general funds of £18,759 to make up the sum distributed. The total funds amount to £1,830,453, and the annual subscriptions amounted to £25,701, showing the public have yet to contribute in this form nearly another £25,000 a year to fulfil the request made by the late King Edward when he issued his original appeal. The donations at the discretion of the Council amounted to over £7,451, whilst the contribution from the League of Mercy was £18,000, a remarkable fact in view of the difficulties which beset all philanthropic effort during 1912. The legacies received were £48,324. It is very interesting and satisfactory to note that the income from investments amounted to no less than £79,278. King Edward estimated that this latter income would amount to £50,000 a year. Providing the public can be made aware that an additional £25,000 a year in annual subscriptions and donations will complete the total revenue asked for at the outset for the Fund, the great work contemplated for it on its inception will be satisfactorily completed.

### THE KING'S FUND AND ST. GEORGE'S HOSPITAL.

At the Council of the King's Fund on the 23rd instant, the report of the Distribution Committee upon the application of St. George's Hospital for a grant was submitted, together with one from the Executive Committee upon the renewed request from St. George's Hospital for an independent inquiry. These matters were fully discussed and the report of the Distribution Committee was unanimously adopted. For the reason just stated it is not possible for us to publish the report of the discussion till our next issue.

### DEATH OF A LONDON HOSPITAL SECRETARY.

We regret to record the death of Mr. W. Guntrip King, Secretary of the Samaritan Free Hospital for Women in Marylebone Road. Mr. King had been suffering from a serious disease for some time, and his work had been interrupted for some months previous to his death, which took place on Saturday last. He had been connected with the Samaritan Hospital for thirty-seven years, entering first as clerk, and later being promoted to the post of secretary. A genial nature and kind demeanour distinguished him, and he belonged, perhaps, to a type of institutional officer which is passing away. During his period of office the new building was opened, in 1890, and additions were made in 1905-7. The funeral took place in the presence of many of his colleagues on Wednesday last at Finchley.

### KING EDWARD VII. CHILDREN'S HOSPITAL, BIRMINGHAM.

On Wednesday last, it was arranged that Princess Louise, Duchess of Argyll, should lay the foundation-stone of the King Edward VII. Children's Memorial Hospital, Birmingham. This was an exceptionally interesting occasion, as it marked the fulfilment of a long-expressed wish for an entirely new and up-to-date children's hospital. The site in Ladywood Road has been paid for, and the committee has in hand and in view £49,000, or £13,000 less than will be required if the new building is to be opened free of debt. The new hospital will contain about 130 beds, and the wards are being built on the open-air principle in such a way as to secure that the whole of the side of each ward shall be open and face south-west. The wards, of course, will be capable of being closed with very little trouble, and the design appears to resemble that of the Liverpool Country Hospital at Heswall in Cheshire. The hospital president, Mr. Edward Cheshire, and the chairman of the committee, Mr. Walter E. Pearson, were presented to Princess Louise, and Mr. Cheshire had the honour of presenting an address on behalf of the hospital. The address made it plain that the new hospital is really a reconstruction of the Birmingham and Midland Counties Free Hospital for Sick Children, of which Mr. A. Hastings has been the secretary, and gave an interesting summary of the hospital's history from 1862, the date of its opening, to the present time. This will be the second important move that the hospital has undergone. The architect of the building is Mr. F. W. Martin, and the builder Lieutenant-Colonel J. Barnsley. We are reminded of the successful designing of the Liverpool Country Hospital, and interesting comparisons will probably present themselves between the two institutions, which, with differences, aim largely at a similar result. The important new institution in Birmingham has had a good send-off, and will carry with it the good will of every grade of institutional worker.

### MENTAL WORKERS AND THE COST OF LIVING.

The proposed increases in salaries and wages of the mental hospital workers under the Metropolitan Asylums Board did not approach much nearer to realisation at the meeting of the managers last Saturday, although the contention that the cost of living had substantially increased of recent years was officially vindicated. The Finance Committee, for example, in their report remarked that the increased cost of living was shown in the contract prices of the managers. Mr. Harold Spender, indeed, put the average increase since 1905 at 25 per cent., which is only a more confusing way of stating that twenty-five shillings only goes as far as a sovereign went eight years ago. A change favourable to the officials seems probable, but the managers' decision has yet to be given. We trust that the matter will be delayed no longer, and that the mental workers affected by the proposed increase, which, as already stated in The Hospital, would amount to some £20,000 a year, will be grateful for Mr. Ecroyd's reminder to the Board that their patience has been much strained, and that they are, not unnaturally, a little restive.

### SIR C. ROSE AND A MEDICAL MORAL.

The sudden death of Sir Charles Rose, M.P., chairman of the Royal Aero Club, and well known in sporting circles, is interesting to medical science from the allegation that has been made as to its being the first instance of a death attributed to flight in an aeroplane. The late baronet, however, was sixty-six years of age, and there was apparently nothing in the flight itself to account for the sudden heart failure which occurred so unexpectedly while he was motoring from the flying ground. Motoring, it will be remembered, was soon accused of causing a variety of diseases after it became at all a popular pursuit, but the moral in this case appears to be that men and women past the prime of life are taking an undue risk in flying, since that mode of progression involves a passage through the air, unprotected at present as the motor travelling is, at a speed to which only the strongest heart and lungs should be subjected.

### FLYING PAY FOR MEDICAL AIRMEN.

According to an Order in Council published last week, medical officers who obtain the certificate of the Royal Aero Club at their own expense are to be eligible for the gratuity of £75, provided that the permission of the Admiralty to undergo such training has first been obtained. Also, medical officers trained at the Central Flying School or at a naval flying school are to be paid half the authorised rate of flying pay while under instruction, and after attaining such proficiency as may be laid down, they are to be paid the authorised rate of flying pay for any days on which they may be required to fly on duty, and for days on which it may be necessary for them to carry out practice flights, not exceeding two per month. The place of the airman in modern warfare is still a matter of dispute; but it is significant that medical officers are receiving encouragement, such as it is, to add to their special function a new proficiency of this kind, which probably their own initiative has won for them.

### SURPRISE GIFT TO A FESTIVITIES FUND.

The decoration of hospital wards at Christmas time may seem a far-off problem in April, but the fund for ward decoration at Nottingham General Hospital has, nevertheless, had a pleasant surprise. For Mr. R. M. Knowles, of Colston Basset, High Sheriff of Nottingham, in reply to a request for evergreens, with which to decorate the wards at Christmas time, has written to say that he could not comply with the request, but he sent with his letter a cheque for £1,000, which he hopes will prove of more permanent advantage. This raises the interesting question as to whether it is good or bad policy to have a small endowment for the expenses of Christmas festivities or not. We think that few of our readers, who remember Mr. J. H. Smethurst's article in our columns describing how the money for festivities was raised locally, through personal effort in varied and original ways, will not agree that personal service each year in this matter is better policy than endowment. For Christmas is a time in which the hospital's supporters are brought into closer touch with the institution than at any other season, and the decorations and entertainments should be the direct product of their affection and enterprise.

### THE INSURED AWAY FROM HOME.

Like many other problems connected with the muddle known as the Insurance Act, the treatment of the insured when away from home has provoked further "modifications" on the part of the Commissioners. Hitherto, upon notice being given by the person removing, there was supposed to be a transference from one committee to another of such proportion of nine shillings as may be gauged by the fraction of the year spent in the new area. Insured persons, however, we learn from the official memorandum of the Commissioners, cannot be induced to give notice unless they are ill, or expect to be ill, at the time of removal. Nor do we blame them when we consider the numerous vexatious regulations which they have passively acquiesced in hitherto. The result of their refusing to be bothered on the present point has been, of course, an insufficient transfer of funds between the areas to defray the cost of temporary residents. The Commissioners, therefore, have been obliged to form a central fund, to be constituted by transfers of funds in respect of "temporary residents" actually attended, whose treatment will be paid for on an attendance basis. The administrative effect of the above modification appears to be to relieve the wretched insured person of one of the many documents he is required to fill up, and to throw on the Commissioners and Insurance Committees the routine of arranging the transfer of money. This is an interesting example of the success which awaits the refusal of people of the insured class to carry out vexatious regulations, and points the way to avoid being bullied by an Act of Parliament apparently designed to harass the poorer classes as much as they will stand.

### LONDON DISPENSARIES AND THE SATURDAY FUND.

In view of the investigations into the food of the working classes at Glasgow, which we recently discussed, Dr. T. D. Lister's arguments in favour of the tuberculosis dispensary for London, which he put forward at the annual meeting of the Hospital Saturday Fund, may receive additional attention. Consumption as a cause of death, he said, was due largely to the triple evils of bad food, bad housing, and bad habits,—the order of which was, no doubt, carefully chosen. The first evil, bad food, the dispensary cannot cope with, for it really means insufficient food, and this, " instruction " can never, of course, remedy. The dispensary's second function Dr. Lister defined as that of the classification of patients in various stages of the disease. These arguments are interesting, because although they are not new, they are examples of the way in which the Hospital Saturday Fund has become the centre of discussions and debates on many sides of institutional life. Mr. Lawson's speech elsewhere referred to is another example, and the interest shown by its members is fruitful for good.

### WORKHOUSE INFIRMARIES WITHOUT THEATRES.

Earlier this month a discussion was opened at a meeting of the Chesterfield Board of Guardians as to whether or not an operating theatre should be provided at the Workhouse hospital. The resolution embodying the proposal was moved by Canon Prior, who pointed out that the board's infirmary ought to be at least as well equipped as those of neighbouring unions. He pointed out also that a theatre would be of use not merely to patients but to the infirmary staff, and, further, that the expense would be small, as a room could be adapted, and the principal expense would be the provision of an operating table, which was expected to cost about £20. We labour these arguments at some length because this inevitable and essential proposal was not allowed to pass without some, however cautious, opposition. It is almost incredible at this time of day that any workhouse infirmary should be allowed to exist without its own operating room, and that any Board of Guardians should be otherwise than determined and eager to be up to date in such an essential provision. The Chesterfield Board of Guardians passed, indeed, Canon Prior's resolution, but, as a matter of record of how slow and painful progress is, we are bound to add that Archdeacon Crosse urged the Guardians to consider the expense involved, on the ground that they would be duplicating what existed already " down the road "—namely, at the Chesterfield Hospital. To consider the expense of any reform must be part of a Board of Guardians' duty, but to imply that an operating theatre at a workhouse infirmary is a duplication (that is, virtually, non-essential) belongs to an age that has passed.

### THE HOSPITAL DOOR.

The importance of a properly constructed hospital door is more fully recognised by those interested in hospital construction than hitherto, for the rapid advance made in the practice of asepsis has been accompanied by a great, if sometimes unnecessary, development of the smaller details of constructional technique. Whilst the ordinary panel door, which offers lodgment for dust and dirt, is still to be seen, even in the more modern hospital buildings, this type is fast disappearing, and in new buildings it is replaced by an entirely flush door. An excellent example of a British-made door of this class is exhibited at the Building Exhibition, Olympia, by Messrs. John White and Sons, Ltd., of Bedford. The foundation of this door is composed of an inner frame of " bone-dry " yellow pine, which is covered on each side with an asbestos composition slab, the surface of which is toothed to receive veneer. The side edges and top and bottom are lined with a solid hardwood fillet. In cases where the highest finish is not required and cost is a consideration, the veneer may be omitted, as the asbestos slab lends itself admirably for painting. The door is fire-resisting and very durable, and combines with these qualities an advantage in construction which renders it almost totally insensitive to changes of atmosphere and temperature. It is interesting to note that these doors have been hung in the new buildings of the Bristol Royal Infirmary.

### BOLTON INFIRMARY AND INSURED PERSONS.

Advantage of the half-yearly meeting of the Bolton Infirmary was taken by Mr. Briscoe to deal with the relation of the institution to insured persons. It was said, he explained, though quite falsely, that the infirmary officials were going round and asking insured persons for their insurance money. They had never asked a patient in the infirmary for his insurance money, and he could not understand why the representatives of approved societies should say that the money for insured persons would go to the infirmary. It was not right that insured persons should be told that they could not receive sick benefit until they left the hospital. There was a resolution in the infirmary books that no such money must be taken. In the face of this it is a matter of importance to bring to book the authors of these allegations, which are all the more wanton because the entry in the books referred to must have been preceded by a discussion of which the local press would have taken note.

### THE LETTER SYSTEM AT WEST HAM.

A very important matter engaged the attention of the committee of the West Ham Hospital last week, when the chairman, Mr. T. A. Cook, moved that " the out-patient letter system be abolished." He quoted the King's Fund Report on out-patients, to the effect that the general opinion was in favour of their abolition, and remarked on the hardship often experienced by patients in getting them, and on the difficulty, under the letter system, of preventing abuse, through the careless giving of letters, without suitable inquiry, to unsuitable cases. After Mr. J. B. Haines had seconded the motion, the case for the letter system was put forward by Mr. J. W. Smith, who said that the giving of letters was much valued by subscribers, whose only privilege it was to exercise the patronage thus conferred. He also expressed the view that the letter system could only be abolished at an annual or special meeting of governors, because the recognition of the system was part of their present by-laws. Mr. Howard Tanner thought that the subscribers' views ought to be heard. The medical staff, whose views were expressed by Dr. Nicholl, were in favour of the Chairman's proposal, on the ground that interesting cases often disappeared because the patient had been unable to get another letter. He added that last year more than a third of the letters sent out were not used. The letter system should not stand in the way of free treatment to those entitled to it. After the chairman had said he thought that the abolition of the letter system would increase the general interest in the hospital, tne motion was put and lost. It was agreed, however, that no applicant for advice at the out-patients' department should be refused if he had no letter, and a committee was appointed to consider the matter. This is the thin edge of the wedge. For Mr. Cook's view is the right one, as may be proved by the fact that where the letter system has never been in operation it has never been missed: the voluntary system is based on something more vital than a love of petty patronage.

### ELECTRIC AMBULANCES FOR LONDON.

The need for electric ambulances in the Metropolis, and by implication in other large cities, was emphasised by Dr. Waldo at an inquest last Saturday. The deceased was found unconscious on the footway of Tower Bridge, which being on the City side enabled an electric ambulance to be telephoned for, and according to the evidence it arrived in six or seven minutes, and Guy's Hospital was reached eight or ten minutes later. Had the deceased been found on the Southwark side of the bridge, as the Coroner pointed out, this electric ambulance would not have been available, and in consequence it would have taken from twenty minutes to half-an-hour to reach the hospital. Dr. Waldo, with his unfailing persistence in such questions of reform as his work offers him an opportunity of furthering, drew the moral that electric ambulances had become a necessity for all parts of London, and the existing electric ambulance in the City deserves the high credit of having created a new need, and Dr. Waldo for having advocated its extension.

### AN AMBULANCE SERVICE FOR LONDON.

The London County Council's scheme for a new ambulance service does not seem to be arousing much enthusiasm among the other local authorities. The Council, as already explained in The Hospital, have decided to apply for Parliamentary powers to inaugurate an ambulance service by utilising the ambulances already in the possession of the Metropolitan Asylums Board, the Port of London Authority, the Borough Councils, and the Boards of Guardians. The Council has now been informed that the Hammersmith Borough Council and the Guardians of the Westminster Union support the proposal, but there is no intimation as to what the other bodies concerned think of it. At a recent meeting of the London County Council Mr. H. L. Jephson called attention to the matter, and urged that the newly elected Council should be given an opportunity of reconsidering the whole question in order that a more effective scheme might be devised. His appeal, however, met with little response from the Municipal Reform majority, and it was intimated that they intended to proceed with their original proposal.

### HOSPITAL TRIBUTE TO SIR H. SWANZY.

A most interesting tribute to the hospital work of the late Sir Henry Swanzy, whose death we referred to last week, is contained in a resolution passed by the council of the Royal Victoria Eye and Ear Hospital, Dublin, to which Sir Henry was honorary secretary. "The hospital," states the resolution, "owes its origin almost entirely to his initiative and public spirit," and the present existing excellence of its structure to his personal attention to every detail. "It was through his loyal and single-hearted efforts that the many difficulties surrounding the effectual amalgamation of the two older hospitals—St. Mark's Ophthalmic Hospital and the National Eye and Ear Infirmary—were surmounted, through the machinery of the Act of Parliament obtained in 1897. This hospital was the great aim of his life, and it stands to-day as a lasting and a worthy monument to his memory." There is no doubt that the late surgeon was an example of one of the best types which the voluntary system produces, and his connection with the institutional side of his hospital is a great tribute to his personal service.

### HOSPITAL REVENUES AND NATIONAL INSURANCE.

We are indebted to Mr. E. C. Smith, the Secretary, for an early copy of the North Ormesby Hospital report for 1912. The feature of this report is an appeal to annual subscribers to contribute twice the ordinary amount during 1913 and to every workman to increase his weekly subscription by a halfpenny for twelve weeks, in order to free the hospital from debt by a collective contribution of £1,753. We shall be glad to hear in due course how far this invitation has been acted upon. The hospital is dependent upon the million and not upon a few rich individual subscribers, and it does a much-needed work for the community which it serves by maintaining upwards of 100 beds. The number of beds occupied fell from 96 in 1911 to 85.62 during 1912, owing to the strike, which lasted two months. It is interesting to note in this connection that the strike increased the average cost for an in-patient per diem by upwards of 4½d. and for each in-patient by 6s., which is equal to an increase in the cost per bed occupied of £7 7s. in round figures. The strike entailed a loss in the income derived from workpeople of £600. Mr. Smith does not anticipate that National Insurance will have any effect upon workpeople's contributions so far as this hospital is concerned, though it may affect the annual subscriptions from individuals. It will not be possible to estimate the actual effects of National Insurance upon the revenues of voluntary hospitals until the audited accounts are ready for the whole of 1913.

### THE FUTILITY OF CLOSING BEDS.

It may be interesting to note, however, that the revenue figures for the above hospital (North Ormesby) for the year 1911-12 show that the annual subscriptions have increased by £8 5s. 1d., the donations by £17 17s., and the employees' subscriptions by £30 10s. 3d., though the Hospital Saturday and Sunday Funds show a falling off of £22 14s. 10d., and the revenue from entertainments has diminished by £20 11s. The annual income for 1912 shows a net increase of £383 8s. 10d. compared with that of 1911, in addition to special donations amounting to £524 7s. towards the liquidation of the debt on the hospital. Although the number of in-patients treated in 1912 is 1,324, compared with 1,410 in 1911, the expenditure only shows a net decrease of £16 17s. 11d. This is a striking proof of the futility of endeavouring to reduce the expenditure of a hospital by closing beds. Closed beds mean a relatively small reduction in the establishment charges of a hospital, a fact which is too seldom recognised. The giving public are beginning to realise that a hospital which threatens to close beds if more money is not forthcoming reveals the fact that its managers lack experience, both in the reduction of cost and in the raising of revenue.

### MEMORIAL TO JOHN HARRISON.

The founder of the London Hospital, John Harrison, who was also its first surgeon, has at last received a memorial other than that which the great institution itself has become. The decision to place a tablet on the wall of St. Paul's Church, Deptford, where he is buried, was previously announced in The Hospital, and we again give the words on the tablet which was unveiled on Sunday, April 13. They are as follows: "In memory of John Harrison, founder and first surgeon of the London Hospital, who died in 1753, and was buried in this churchyard. His body lies here. His work continues at the hospital." The admirable terseness of this epitaph should be an example to all who have to devise similar memorials.

### THE CUMBERLAND INFIRMARY APPEAL.

It must be very gratifying to Dr. Henry Barnes, consulting physician to Cumberland Infirmary, to report the response to the recently issued appeal which he himself has done so much to further. If, however, the total of £800, at present received, of which one-third consists of new or increased annual subscriptions, is insufficient to meet the needs of the institution, as it certainly is, it is very satisfactory to note the way in which individual members of the working classes have come forward and subscribed according to their means. For though the sum total from the poorer members of society must necessarily be slender, the goodwill thus shown is widespread, and it is upon the spread of such goodwill that voluntary finance in the end depends. Business and charity join hands in proving that it is on an aggregate of small sums rather than in large gifts, that success in every sense depends. All honour to Dr. Barnes for setting the stone rolling.

### THE REOPENING OF CIRENCESTER HOSPITAL.

We are glad to record the reopening last week of the Cirencester Cottage Hospital, which has been closed for the building of a new wing. It is probably the best administered cottage hospital in the country, and the medical surgical and nursing work is of the very best a modern hospital can produce. This addition, which doubles the size of the institution, is the work of Mr. V. A. Lawson, architect, and has been carried out at a cost of £2,500, the money for which was generously provided by the late Mr. D. G. Bingham. The beds now number twenty or more, and the hospital provides another example of the way in which the best cottage hospitals grow and develop from small beginnings into institutions to which the term "cottage" in the end no longer applies. We are glad to note also that a cheque from Mrs. Wilfred Cripps for £500 was handed by Lord Bathurst to the Treasurer, to be added to the Endowment Fund, and we hope that the increased usefulness of the institution will bring not merely more patients but more maintenance funds to the enlarged hospital.

### DIPHTHERIA OUTBREAK AT COVENTRY HOSPITAL.

An interesting statement on the outbreak of diphtheria at the Coventry and Warwickshire Hospital was made by the Chairman, Mr. E. M. Iliffe, at the general committee meeting last week. Since January 15, he said, 15 patients had been sent to the hospital for operation. Of these 15, 12 had been operated on, and only 10 of the 15 patients were injected with serum before coming to the hospital. So that in five of the cases the conditions agreed upon with the city authorities and the hospital were not observed. On February 13 the senior house surgeon performed the operation of tracheotomy on one of the fifteen patients, and a couple of days afterwards he developed symptoms of diphtheria and was isolated. Eventually swabs were taken from the throats of all the patients. Diphtheria bacilli were found in no less than thirty-four cases of the patients and members of the hospital staff; twenty-four of these were removed to the fever hospital, of which nineteen proved to be merely germ-carriers. The other ten cases of the thirty-four were retained in the hospital, and in three of them the patients died from diseases other than diphtheria. Swabs were taken and sent to the Lister Institute, and came back marked "positive" after the patients had unfortunately died. One of the ten patients was still in the hospital in an isolation ward, and all the other six had been discharged, either as cured or not having developed the disease. So far as the hospital was concerned the state of affairs was not serious. They had taken precautions, and because the precautions were rather exceptional it might lead the public to believe that the hospital was infected with diphtheria. As a matter of fact it was not. The only bad cases were cases which were sent there for treatment. The new kitchen has started work, and the two new wards were due for occupation this week.

### DR. AGNES McLAREN'S DEATH.

The death of Dr. Agnes McLaren removes another of those honoured pioneers who, with the late Dr. Jex-Blake, won the battle for the medical education of women. The story of their agitation carries one back to the heroic days at Edinburgh, and it was, in fact, at the house of Dr. McLaren's father that Miss Jex-Blake and her fellow enthusiasts, Miss Peachey among them, used to meet. The opening of the medical schools, including the clinical wards of the Edinburgh Royal Infirmary, was, of course, bitterly opposed by the medical students and others, and we need not repeat once more the lengthy story which was crowned with success at Edinburgh and London in the end. Dr. Agnes McLaren died at Cannes, where she had long enjoyed a specialist practice, though of late years she devoted her attention to active work against the White Slave Traffic, and on behalf of various social reforms. Philanthropists of every class knew and esteemed her, and her reception into the Roman Catholic Church led to her enlisting the active support of the Vatican in her projects.

### PHYSICAL EXERCISES IN SCHOOLS.

Following on the article in The Hospital of April 12 on "Physical Exercises in Schools," there was a discussion on the subject at the meeting of the London Education Committee on April 16. Major Levita called attention to the question, and said that not through any fault of the teachers, but possibly through the need of looking into the system, the physical training of the children of London was not being carried out in the way it ought to be. No two of the schools had a similar form of carrying out the exercises. He thought that training was done in a perfunctory manner. They required somebody not of the drill-sergeant type to supervise the work, not only of the children but of the teachers, somebody who was in touch with the latest methods of Swedish training. Mr. W. A. Clague said that the syllabus was laid down by the Board of Education after consultation with a military man of repute and educationists throughout England. The syllabus was perhaps a little difficult, but teachers had discretion in using it. Our article made it abundantly clear how necessary was expert supervision, and how evil may be the results of exercises, supplied without discretion, which is a quality that expert training alone can secure.

### ALLEGED SCARCITY OF SHIPS' SURGEONS.

If rumours be correct, the charm of the ship surgeon's life is proving insufficient to attract sufficient medical men, and the steamship companies are reported to be complaining. In other words, more vacancies for ship surgeons' posts are reported than there are applicants; so, here again another of the medical services has been discovered which is not over-crowded! What is the reason for this? It must be put down partly to the fact that the competition for a practice is now so great that many medical men hardly feel that they can afford to be out of the stream, and that the post of ship's surgeon as such does not lead directly anywhere. Partly, in spite of statements to the contrary, the attractions must be held insufficiently great. But, so long as human nature needs a holiday, and purses are not overfilled, the post of ship's surgeon must continue to offer advantages, of the temporary kind required for this purpose, as in many senses the ideal holiday for medical men.

### LONDON'S PUBLIC HEALTH COMMITTEE.

The Public Health Committee of the London County Council have appointed Mr. F. R. Anderton to be their chairman during the coming year, and Mr. Gilbert Johnstone to be their vice-chairman. Mr. Anderton, as chairman of the Children's Care Sub-Committee of the Education Committee, was largely responsible for the inauguration of the Council's arrangement with the hospitals for the medical treatment of school children. Mr. Johnstone was formerly chairman of the Midwives Act Committee. The work of the Public Health Committee will be specially interesting during the coming year, as they are at present engaged in preparing a comprehensive scheme for the treatment of tuberculosis in London.

### THIS WEEK'S DRUG MARKET.

Very little improvement has taken place in business during the past week, and the Drug market is uncommonly quiet for the time of the year. Interest in cod-liver oil has abated and prices are still inclined to recede in spite of the poor results of the fishing; there is a point, however, below which the price of the article is not likely to fall in view of the curtailment of supply, but buyers would perhaps be well advised to wait for a week or two before fulfilling their requirements. The yield of oil up to the present this season is half that of last season's, but almost the same as the yield up to the same date in 1911. At this time in 1911 the value of cod-liver oil was considerably higher than the present value, and any further reduction in quotations for this season's oil can hardly be very large. Oil of almonds is slightly dearer. Balsam copaiba is tending upwards in price, and opium is also inclined towards higher prices. There has been no further change in the price of cocaine hydrochloride, but any alteration would be likely to be in a downward rather than an upward direction. Sugar of milk is slightly cheaper. At the recent public sales in Mincing Lane. Cape aloes sold at dearer rates, but asafetida and menthol tended lower.

